# Influence of montane altitudinal ranges on species distribution models; evidence in Andean blow flies

**DOI:** 10.7717/peerj.10370

**Published:** 2020-12-07

**Authors:** Mariano Altamiranda-Saavedra, Eduardo Amat, Luz Miryam Gómez-P

**Affiliations:** 1 Grupo de Investigación Bioforense, Facultad de Derecho y Ciencias Forenses, Tecnológico de Antioquia Institución Universitaria, Medellín, Colombia; 2 Grupo de investigación GESTAS, Politécnico Colombiano Jaime Isaza Cadavid, Medellín, Colombia

**Keywords:** Regionalization, Human influence index, Species distribution models, Synanthropy

## Abstract

**Background:**

Blow flies are a family of dipterans of medical, veterinary and sanitary importance. We aim to predict the current geographical distribution of six neotropical blowfly species with different altitudinal ranges of distribution (high, medium, and lowlands) and degree of synanthropy (eusynanthropic, hemisynanthropic and asynanthropic) based on their existing fundamental niche (E_A_) in Northwestern South America.

**Methods:**

Geographical records were compiled based on data from museum specimens and literature. The accessible area hypothesis (M) was calculated based on three criteria: (1) Altitudinal range, (2) Synanthropy values deducted based on the Human Influence Index (HII) raster dataset, and (3). The mean dispersal capability of flies. The modeling was performed using the Maxent entropy modeling software. The selection of parameters was made with the R Program ENMeval package.

**Results:**

The models were assessed using the area under the operator-partial receiver curve (ROCp). The high statistical performance was evidenced in every modeling prediction. The modeling allowed identifying possible taxonomic inaccuracies and the lack of exhaustive collection in the field, especially for lowlands species. Geographical distribution predicted by the modeling and empirical data was remarkably coherent in montane species.

**Discussion:**

The data obtained evidence that montane elevational ranges affect the performance of the distribution models. These models will allow a more precise predicting of medium and high elevation blow flies than lowlands species. Montane species modeling will accurately predict the fly occurrence to use such biological information for medical, legal, veterinary, and conservation purposes.

## Introduction

Blow flies (Calliphoridae, Oestroidea) are a family of dipterans consisting of approximately 1,600 species worldwide (Pape, Blagoderov & Mostovski, 2011), and approximately 100 occurring in the Neotropical region (Kosmann et al., 2013). Most carrion-feeding species are of medical, veterinary forensic and sanitary importance. They are of sarcosaprophagous habits and are among the most active scavengers of the food chain, reducing dead organic matter or waste from primary producers to consumers (Galante & Marcos-Garcia, 1997). Most species are strongly attracted by volatile compounds emanated by feces, secretions, decaying materials, and food. This flying tendency between rotten materials and human comestibles makes them efficient mechanical vectors of pathogens (Greenberg, 1973). Adults have a well-developed olfactory system that is very sensitive to carrion and allows them to locate it at great distances quickly (Yan et al., 2018). Female flies lay eggs (some species deposit larvae) on the decaying material and began the development; larvae are the most important organisms in the carcass reduction process (Anderson & Cervanka, 2002), determining its age is used in forensic entomology as a biological clock to calculate the postmortem interval (PMI) (Greenberg & Kunich, 2002). Some species can also develop in animals and humans’ wounds, where they feed on living tissue, producing myiasis (infestation of alive tissue by maggots) (Norris, 1965; Francesconi & Lupi, 2012).

The spatial distribution of flies fundamentally depends on tolerance to environmental conditions, being affected by the degree of human impact (anthropization process), as natural landscapes become urbanized (Kavazos & Wallman, 2012). In entomological studies, the anthropic level of preference is commonly referred to as “synanthropy” (Gregor & Povolný, 1958). Thus, synanthropic, hemisynanthropic, and asynanthropic are categories for classifying flies based upon their degree of attraction or repulsion to human settlements (Povolný, 1971). This ecological classification for Necrophagous Diptera has been widely used to understand spatial patterns for habitat association or an urban-rural gradient (Nuorteva, 1963; Hwang & Turner, 2005; Kavazos & Wallman, 2012; Amat & Medina, in press). Potential spatial distribution by modeling an ecological niche has become a frequent subject of study (Austin, 2002). In order to accurately assess the geographical distribution of organisms, several methodologies have been proposed (Anderson, Lew & Peterson, 2003; Qiao et al., 2016a; Qiao et al., 2016b). Models help locate areas of suitable environmental for species thriving and settling based on parameters previously obtained from the field (Peterson et al., 2002). Modeling was useful in assessing distributional and geographical patterns in biogeographical, ecological and conservational contexts (Anderson, Lew & Peterson, 2003).

The use of these models has become popular in recent years, mainly for the study of plants and vertebrates such as mammals, birds, amphibians, and reptiles, and on a smaller scale, of invertebrates and insects (Elith & Leathwick, 2009; Peterson et al., 2011; Peterson et al., 2020; Qiao et al., 2016a; Qiao et al., 2016b). Few studies evaluate the relationship between the spatial distribution of blowfly species and their environment using species distribution models (SDMs) (Lecheta, Corrêa & Moura, 2017; Richards, Williams & Villet, 2009; Mulieri & Patitucci, 2019). In South Africa, the potential distribution of seven important blow flies was analyzed, and it was found that the prediction of the distribution of restricted species was more accurate than those more widespread in the region (Richards, Williams & Villet, 2009). Later, Williams, Richards & Villet (2014) discovered no correlation between species distributions and sheep farms, or human populations. In South America, the potential geographic distribution of the blow fly *Sarconesia chlorogaster* (Diptera: Calliphoridae) was estimated, and it predicted suitable areas in Ecuador and Colombia, which had no previous records (Lecheta, Corrêa & Moura, 2017). Recently, the potential distribution of *Cochliomyia hominivorax* (Diptera: Calliphoridae) was estimated, and it was determined that large areas in the central-eastern territory of Argentina are environmentally suitable for potential occurrence, and the seasonal temperature fluctuation is the most significant contributor to explain such distribution (Mulieri & Patitucci, 2019).

We aim to evaluate if the altitudinal range influence the current potential geographical distribution of six neotropical blowfly species with different altitudinal ranges of distribution (high, medium and lowlands) and different degree of synanthropy (eusynanthropic, hemisynanthropic and asynanthropic) based on their existing fundamental niche (*E*_A_) in Northwestern South America by considering the accessible area hypothesis (M) calculated based on three criteria: (1) Altitudinal range, (2) Synanthropy values are deducted based on the Human Influence Index (HII) raster dataset, and (3) The mean dispersal capability of flies. This contribution will facilitate the use of ecological and chorological data of flies for medical-legal, veterinary, and conservational purposes.

## Materials & Methods

### Study area

**Figure 1:**
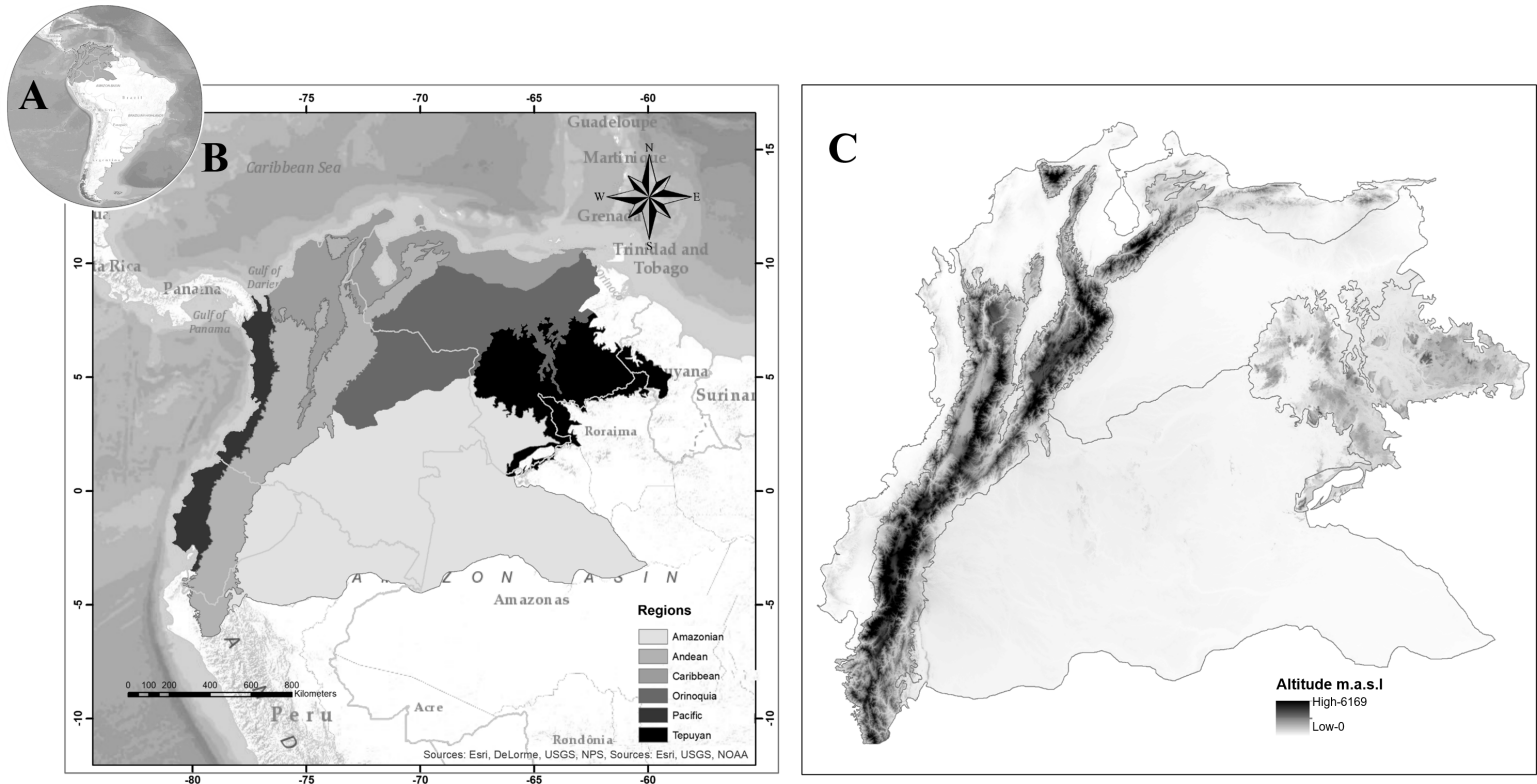
Map of Northwestern South America, (A) Study area in South America, (B) proposed regionalization for blowfly fauna (Diptera: Calliphoridae), (C) altitude in the study area.

The area is located in the northwestern extreme of South America; it includes the political borders of five countries: Northwestern Brazil, Northern Peru, Colombia, Ecuador, and Venezuela. In a biogeographical sense, it is possible to differentiate six natural regions. The blowfly spatial distribution follows the biogeographical regionalization proposed in Amat (2017) (Fig. 1).

### Presence data

After disposing of spatial autocorrelation with the R package ntbox (Osorio-Olvera et al., 2020), the complete occurrence database included 228 records of presence, including asynanthropic and medium lowland (1,100–2,600 m.a.s.l) distribution *Blepharicnema splendens* (Diptera: Calliphoridae) (22) and high land (2,000–3,127 m.a.s.l) distribution *Sarconesia roraima* (Diptera: Calliphoridae) (15); hemisynanthropic and lowland (0–1,000 m.a.s.l) *Chloroprocta idioidea* (Diptera: Calliphoridae) (59) and medium lowland (1,100–2,700 m.a.s.l) distribution *Lucilia purpurascens* (Diptera: Calliphoridae) (30); eusynanthropic and high land (2,200–2,800) distribution *Calliphora vicina* (Diptera: Calliphoridae) (19) and lowland (0–1,400 m.a.s.l) *Cochliomyia macellaria* (Diptera: Calliphoridae) (83) (Table S1). Presence records by species were obtained from the geographical information on specimens revised in eleven entomological museums, and recent literature on blow flies published since 2000. Data for each specie were organized as presence records in a database, with their coordinates in decimal degrees projected in the WGS84 system.

Acronyms of museums, collections and depositaries of specimens are as follows: MIZA Museo del Instituto de Zoología Agrícola, Francisco Fernández Yepes. Universidad Central de Venezuela, Maracay, Venezuela. INPA Coleção de Invertebrados, Instituto Nacional de Pesquisas da Amazônia, Manaus, Brazil. MECN Museo Ecuatoriano de Ciencias Naturales. Quito, Ecuador. QCAZ-I Museo de Zoología - Sección Invertebrados, Facultad de Ciencias Exactas y Naturales, Pontificia Universidad Católica del Ecuador, Quito, Ecuador. IAVH-E Colección entomológica - Instituto de Investigaciones Biológicas Alexander von Humboldt, Villa de Leyva, Boyacá, Colombia. ICN-MHN Instituto de Ciencias Naturales –Museo de Historia Natural, Facultad de Ciencias, Universidad Nacional de Colombia. Bogotá, Colombia. CETdeA Colección entomológica del Tecnológico de Antioquia, Institución Universitaria, Medellín, Colombia. MEFLG Museo Entomológico Francisco Luis Gallego, Universidad Nacional de Colombia, sede Medellín, Medellín, Colombia. UNAB Museo Entomológico, Facultad de Agronomía, Universidad Nacional de Colombia, sede Bogotá, Colombia. UPTC Museo Luis Gonzalo Andrade. Universidad Pedagógica y Tecnológica de Colombia, Tunja, Boyacá, Colombia.

### Environmental layers

The variables used to characterize the existing fundamental niche of the species of interest and evaluate their potential distribution were obtained from Worldclim, version 2.0, (spatial resolution = 30 arcseconds, ∼1 km) These are derived from monthly values of precipitation and temperature with biological significance and represent annual trends of temperature, precipitation, seasonality, and extreme or limiting environmental factors (Fick & Hijmans, 2017) . In order to reduce collinearity in environmental layers, a Pearson correlation analysis was conducted with the tool known as “SDMtoolbox” on ArcGis 10.3. Variables with correlation value >—0.8— were eliminated (Raghavan et al., 2019). Additionally, we used the Jackknife option from the Maxent software to identify variables that did not provide a significant contribution to the robustness of the models.

### Model design

A key element in the development of ecological niche models is the hypotheses of areas (M) that have been accessible to the species in relevant periods (Barve et al., 2011). With the available information on the natural history and biogeography of the species of interest, we created hypotheses for the accessible areas (M) by establishing different criteria: (1) reclassifying a digital elevation model (DEM) from Shuttle Radar Topography Mission (SRTM) (Rabus et al., 2003) with the information on altitudinal range reported for species (*B. splendens* 1,000–2,700 masl, *S. roraima* 1,500–3,500 masl, *L. purpurascens* 1,000–3,000 masl, *C. vicina* 1,500–2,500 masl, *C. macellaria* 0–2,000 masl and *C. idioidea* 30-1200 masl; 2) the value of synanthropy was measured using a raster from the Human Influence Index (HII) dataset of the Last of the Wild Project, version 2 (Wildlife Conservation Society WCS & University C for IESIN-C-C, 2005). The dataset integrates information from nine layers related to human population densities, land use, infrastructure, and human transportation access. As for asynanthropic species, we took into account the maximum value of this index for occurrence data, the minimum value for eusynanthropy, and finally, the range for hemisynanthropy; (3) the result of the mask of altitude and index of synanthropy was added to a buffer with dispersion capacity data of the species measured in Km2. For species lacking this information, we used the minimum known flight range value for the Calliphoridae family (4.8 km) (Bishopp & Laake, 1921; Lindquist et al., 1951; Quarterman, Mathis & Kilpatrick, 1954; Shura-Bura et al., 1958).

We assessed the current geographical distribution based on the existing fundamental niche concept (E_A_) (Peterson et al., 2011), using Maxent 3.3.3k (Phillips, Anderson & Schapire, 2006) to estimate environmental suitability in these analyses. To assess the optimal parametrization of the suitability estimates in the calibration region, different settings were tested using the ENMeval package of the R program (Muscarella et al., 2014), which provides an automated method to execute MaxEnt models across a user-specified range of regularization multiplier (RM) values and feature combinations (FCs) (Muscarella et al., 2014). Then, we set the RM range from 0.5 to 4.0 with 0.5 increments and three FCs, i.e., linear (L), linear and quadratic (LQ), linear, quadratic and product (LQP), linear, quadratic, product and threshold (LQPT) linear, quadratic, product threshold and hinge (LQPTH), resulting in 45 possible combinations of features and regularization multipliers (Muscarella et al., 2014). Output format (row), number of replicates (10), percent used for testing (25%), type of validation (bootstrapping), maximum number of iterations (5000), convergence threshold (0.00001), and maximum number of background points (10,000), no clamping or extrapolation were fixed for each Maxent run.

The fine-tuned MaxEnt models were made by finding the lowest delta value of Akaike’s information criterion corrected for small sample sizes (AICc) among candidate models, which reflects both models, goodness-of-fit and complexity, providing the most conservative results (Basanta, Rebollar & Parra-Olea, 2019) (Table 1). Using the R package ntbox (Osorio-Olvera et al., 2020), a ROC (Receiver Operating Characteristic) was estimated to assess the performance of the model (Peterson, Papes & Sober, 2008). The model was assessed through calibration, randomly selecting 25% of the distribution data and comparing it with the threshold of the area under the curve (AUC). The medians were used through repetitions as a final niche estimate. We established a threshold to convert the raw map of the output of the continuous model into binary assumptions of suitable versus not suitable, using the threshold of lower training presence (LTPT) (Pearson et al., 2006) under an allowable error rate of E = 5%.

**Table 1:** Parameters, set environmental variables used in each model and results of partial ROC analysis (mean value for AUC ratio) to test statistical significance of ecological niche model predictions. A value of 1.0 is equivalent to the performance of a random classifier. These results were based on 100 bootstrap replicates, and statistical significance was assessed via bootstrapping and comparison with a random classifier ratio of 1.0.

**Species**	**Records**	**Environmental variables-relative contribution (%)**	**Feature classes**	**Regularization multiplier**	**ROCp**
*Blepharicnema splendens*	22	bio1 (69.9), bio2 (9), bio8 (1.9), bio12 (1.9), bio14 (1.8), bio15 (3.7), bio19 (10.8)	LQHPT	2	**1.840**
*Sarconesia roraima*	15	bio3 (3.7), bio6 (26.8), bio7 (43.2), bio12 (16.9), bio14 (0), bio15 (3.3), bio16 (0.1), bio19 (3.9)	LQHPT	1.5	**1.924**
*Chloroprocta idioidea*	59	bio1 (0.3), bio6 (14.5), bio7 (23.2), bio9 (5.6), bio12 (0), bio14 (14.5), bio15 (0), bio16(30.6), bio19 (21.9)	LQ	0.5	**1.502**
*Lucilia purpurascens*	30	bio1 (0.9), bio2 (5.1), bio3 (4.4), bio9 (27), bio12 (0.6), bio15 (3.7), bio16 (39), bio19 (36)	LQHPT	1.5	**1.675**
*Calliphora vicina*	19	bio2 (2.5), bio3 (3.6), bio6 (8.4), bio7 (0.9), bio14 (3.4), bio15 (12.2), bio19 (54.5)	LQ	0.5	**1.979**
*Cochliomyia macellaria*	83	bio1 (8.8), bio3 (7.4), bio6 (0), bio8 (2.8), bio9 (6.4), bio12 (18.8), bio14 (10.9), bio15 (30), bio19 (9.3)	LH	1.5	**1.665**

**Notes.**

Bio1 Annual Mean Temperature Bio2 Mean Diurnal Range Bio3 Isothermality Bio4 Temperature Seasonality Bio5 Max Temperature of Warmest Month Bio6 Min Temperature of Coldest Month Bio7 Temperature Annual Range Bio8 Mean Temperature of Wettest Quarter Bio9 Mean Temperature of Driest Quarter Bio10 Mean Temperature of Warmest Quarter Bio11 Mean Temperature of Coldest Quarter Bio12 Annual Precipitation Bio13 Precipitation of Wettest Month Bio14 Precipitation of Driest Month Bio15 Precipitation Seasonality Bio16 Precipitation of Wettest Quarter Bio17 Precipitation of Driest Quarter Bio18 Precipitation of Warmest Quarter Bio19 Precipitation of Coldest Quarter

## Results

**Figure 2:**
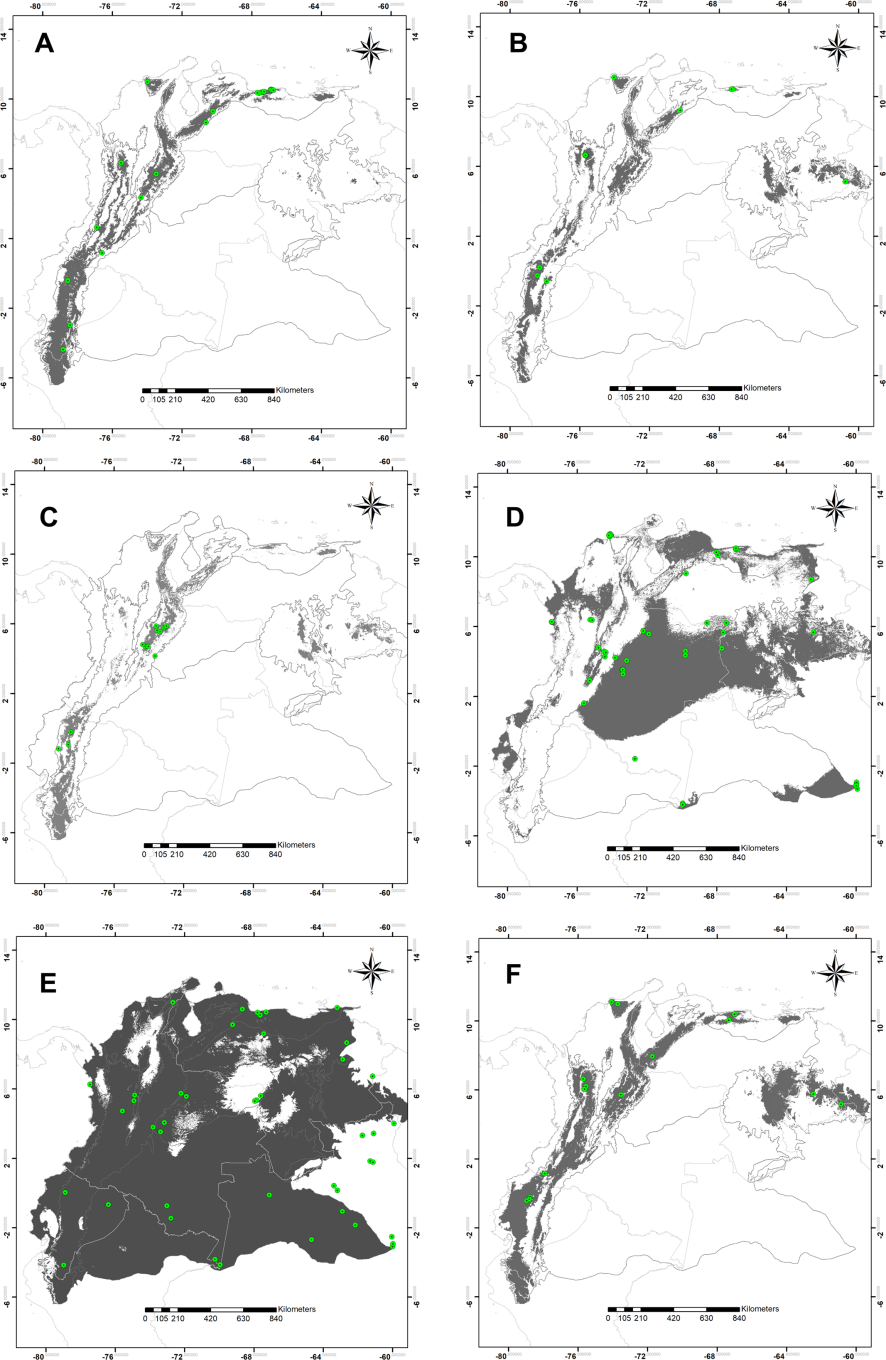
Potential distribution maps. Potential distribution maps of (A) *Blepharicnema splendens*, (B) *Sarconesia roraima*, (C) *Calliphora vicina*, (D) *Cochliomyia macellaria*, (E) *Chloroprocta idioidea* and (F) *Lucilia purpurascens*. Green circles indicate the locations of the records used for calibrating the model; gray areas are modeled suitable conditions; and white areas are unsuitable conditions. Maps at .kml are available for download in the Supplemental_Information.

The high statistical performance was evidenced in all modeling predictions (ROCp > 1.50) (Table 1). Suitable areas for species of montane distribution (*B. splendens*, *C. vicina*, *L. purpurascens*, and *S. roraima*) were associated with elevations; the Andes, Central Cordillera, and tabletop Tepuis in Venezuela. Remarkably, the modeling of potential distribution for *B. splendens* showed suitability areas associated with the Andean and Caribbean regions. These fitting areas correspond to montane ranges along the Andes chain in Peru, Ecuador and Colombia, including the Sierra Nevada de Santa Marta, the Mérida cordillera and the coastal range in Venezuela, but not reaching the tepuis (Fig. 2C). Modeling for *S. roraima* showed large suitable areas uninhabited within the Tepuyan province in the Guyana shield (Fig. 2B). The smallest potential area predicted was obtained for *C. vicina* modeling in the Andean cordillera from Peru to Venezuela, the Cordillera Central, and Venezuelan Tepuis (Fig. 2C). Modeling prediction for *L. purpurascens* was similar to the previous species, including montane regions of the Andes, Pacific, Caribbean, and Tepuis of Venezuela (Fig. 2F). On the other hand, the modeling of *C. macellaria* showed a preference for lowlands areas of the Pacific, Orinoquía, and Amazonia regions (Fig. 2D). Finally, the model for *C. idioidea* with suitable areas was remarkably extended, almost occupying the entire area of study, except for some areas in the Colombian and Venezuelan Orinoquía, and a particular region of the Pacific and Caribbean of Colombia (Fig. 2E).

## Discussion

In carrion-flies ensembles had been demonstrated that the seasonality and the habitat and resource availability are critical aspects of the species occurrence, especially in temperate regions (Smith & Wall, 1997; Martínez-Sánchez, Rojo & Marcos-García, 2000; Arias-Robledo, Stevens & Wall, 2019). Here we focus on the latter aspect since the study area assessed has uniform weather, typical of tropical environments (with dry and wet seasons only). The selected blow fly species have different biogeographical histories (Rognes, 1997). One group belongs to lowland neotropical species of wide distribution related to warm climates, *C. macellaria* and *C. idioidea* (James, 1970); while the second group belongs to temperate species linked to montane medium and high elevations; *C. vicina*, *B. splendens, L. purpurascens*, *S. roraima* (Dear, 1979; Amat, Vélez & Wolff, 2008; Whitworth, 2014). The spatial distribution modeling generated for montane species matches the altitudinal ranges of suitable settlement except for *C. idioidea*. Being the range altitude an essential element for modeling at these latitudes; this proves that elevation is an indirect variable that may provide a good surrogate for temperature across certain extents and latitudes (Austin, 2002). Montane insects are a highly specialized group with particular ecophysiological adaptations to living in extreme altitude environments (Mani, 1968; Hodkinson, 2005). One adaptation is cold-stenothermy, related to narrow thermal requirements to optimal growth. This condition limits the spatial distribution (Hodkinson, 2005), similarly to blow flies montane distributions displayed in this study. This analysis encompasses the environmental and ecological requirements mentioned above, deemed essential for the definition of distribution. However, we found no specific association between the potential distributions of species with a specific bioclimatic variable. The importance of particular environmental variables for a species may vary according to geographic and biotic contexts (Peterson et al., 2011), as evidenced with the modeling for blow flies.

Potential distribution prediction for *B. splendends* matches preliminary observations and occurrences as an endemic Andean taxon distributed from Bolivia to Venezuela made by Amat & Wolff (2007), they reported it as rare and asynanthropic in the Andes inhabiting well preserved forest up to 2,500 m in altitude. The average annual temperature was the most critical variable in explaining the model. This result may suggest that a montane environment with a temperate climate and well-preserved areas under any conservation category (e.g., national park, reserves, sanctuary, and refuges.) may represent suitable conditions for *B. splendens* in the northwestern South America region.

Distribution modeling for *S. roraima* showed a similar montane pattern, being more restricted according to its altitudinal range along the Andean chain reaching Venezuelan Tepuis. *S. roraima* is recorded from Chile at an elevation of 3,250 m (Dear, 1979). In Venezuela, it was reported in the Tepuis, Mérida cordillera, and the coastal range at an elevation of 1,900–2,700 m (Velásquez et al., 2017). It was recorded in Colombia in the central and the eastern ridge at an elevation of 1,900 to 2,592 m (Amat, 2009; Wolff & Kosmann, 2016). Asynanthropic in the surrounding montane areas of Bogotá, Colombia (Pinilla-Beltran, Segura & Bello, 2012). Thus, additional records are expected in the Andean region above 1,900 m in a well-preserved forest, avoiding anthropogenic environments or highly disturbed environments at both Andean slopes in Ecuador and eastern slopes in Peru, in the three Andean ranges of Colombia, including the Sierra Nevada de Santa Marta, and in Venezuela in the Mérida cordillera. Similarly, it is expected to be in the Caribbean region of Venezuela related to the coastal range, especially near the Henri Pittier National Park and elevation ranges at Cueva del Guácharo National Park; and finally, in the Tepuyan region up to Pico da Neblina National Park in Brazil.

*L. purpurascens*, one of the distinct species of *Lucilia* in the New World region (Whitworth, 2014), showed similar montane prediction, including areas above 1,100 m (Fig. 2F) distributed from Argentina to Mexico (James, 1970). In the South American Andes, it is relatively common at an elevation of up to 2,200 m (Whitworth, 2014). Along the cordillera in Peru, it was a rare fly, hemisynanthropic, and ranged 1,300–1,900 m (Baumgartner, 1988). Also, it is known inhabiting the central range in Colombia, up to 2,800 m (Wolff & Kosmann, 2016). It was reported reaching the Páramo ecosystem as an uncommon fly at approximately 3,000 m but is commonly related to forested and rural areas (Amat, 2017). Additionally, it was recorded in the Mérida cordillera and the coastal range up to 2,000 m in Venezuela (Velásquez et al., 2017). The current distributional pattern described above is positively reflected in the modeling obtained.

*C. vicina*, a cosmopolitan species, is strongly associated with urban locations (Rognes & Whitworth, 2012), and cold temperatures (Greco, Brandmayr & Bonacci, 2014). Due to the high degree of synanthropy in the Andean region, it is expected to occur in medium and large cities along the Andean mountain chain above 2,200 m, such as La Mesa (Trujillo), Mérida (Mérida), Tovar (Mérida), San Cristóbal (Táchira), Ocaña (Santander), Pamplona (Norte de Santander), Sogamoso (Boyacá), Tunja (Boyacá), Bogotá, Pasto (Nariño), Ipiales (Nariño), Ibarra (Imbabura), Quito (D.C.), Ambato (Tungurahua), Riobamba (Chimborazo), Cuenca (Azuay), and Loja (Loja). In the Caribbean region, it is expected in Caracas (D.C.) and within a considerable area in the province of Sucre. Finally, in the Tepuyan region, despite the modeling showing relatively suitable environmental conditions, doubts regarding its occurrence raised since no large urban places exist for their settlement. This species has been recorded in the low and intermediate elevations of Chile and Argentina. However, in Northwestern South America, it was collected in high elevations in the Andean region related to cold and large cities in Ecuador and Colombia (Amat, 2017). Pinilla-Beltran, Segura & Bello (2012) reported it as eusynanthropic in Bogotá at an elevation of 2,600 m. In temperate latitudes *C. vicina* is a year-round species, more commonly collected in the winter (Zabala, Díaz & Saloña Bordas, 2014; Arias-Robledo, Stevens & Wall, 2019). Based on South American records from Argentina and Chile, *C. vicina* seems to extend the elevation range upward as it approaches the Equator line (Rognes & Whitworth, 2012), following an occupancy tendency related to cold temperature, as evidenced by the model.

The potential distribution map for *C. macellaria* shows extensive suitable areas in the Amazonian region of Colombia, Venezuela and Brazil, the Orinoquía and inter-Andean valleys of Colombia, the Pacific region of Colombia and Ecuador, the Caribbean region of Colombia and Venezuela, and finally, in low elevations areas surrounding the Venezuelan Tepuis. This wide distribution matches the occurrence pattern mentioned by Dear (1985). Baumgartner & Greenberg (1985) stated that *C. macellaria* was presumably the most common blowfly in the neotropical region before the arrival of exotic *Chrysomya* spp. They referred to this species as premontane under 2,450 m in the central Andes of Peru, similar to Colombia, where it is spread across its five natural regions in forested, rural and urban areas (Amat, 2009). Velásquez et al. (2017) recently cited this species occurring in northern Venezuela up to 1,100 m. Despite *C. macellaria* being classified as eusynanthropic in Peru (S.I = +79) (Baumgartner & Greenberg, 1985) and Colombia (S.I = +69) (Montoya, Sanchez & Wolff, 2009), it is also undoubtedly related to rural and well-preserved environments (hemisynanthropic), as evidenced here by some collection records used for the modeling. Besides, to the suitable areas mentioned above, we expect its occurrence beyond these borders, including a larger portion of the Brazilian, Colombian, Ecuadorian and Peruvian Amazon, a larger area of the Orinoquía in Venezuela and the montane regions ranging up to 2,200 m. These results evidence the lack of exhaustive collection at these sites.

A puzzling result for *C. idioidea* modeling was evidenced since it is usually related to well-preserved rainforests in lowlands (Baumgartner, 1988; Amat, 2009). This distributional pattern contrasts with suitable areas here reflected (Fig. 2E), which includes large regions of montane and highlands environments (e.g., high Andean forest and Páramo) exceeding their expected altitudinal range. Several montane records above 1,000 m were included to build the model; these records belong to polymorphic specimens with the pattern of body and wings coloration being darker. *C. idioidea* was initially considered as a species complex (Shannon, 1926; Hall, 1948). However, Dear (1985) mentioned a notable variation of coloration pattern depending on the geographical distribution but still considered it a single species. Moreover, in Peru, Baumgartner & Greenberg (1985) noticed two types of phenotypes differing in anterior facets’ size. Yusseff-Vanegas & Agnarsson (2017) in recent molecular studies based on mitochondrial (COI) and nuclear (ITS2) DNA sequences, in specimens from Central America and the Caribbean indicate at least the existence of two species in *C. idioidea*, they also evidenced some morphological differences. Morphological differences here detected and the extended distribution pattern predicted, led us to believe that the current model was probably performed based on at least two different life-histories. We suspect that montane specimens here reviewed may be part of a cryptic species of montane distribution, contrasting the taxonomic arrangement of Dear (1985). Caution is suggested to use this modeling since the current taxonomic status of *C. idioidea* and its populations must be revised.

## Conclusions

In general, our results showed that montane distributional patterns affect the performance of SDMs in neotropical blow flies. Montane species models fit better with the known current distribution and the biogeographical region (Andean province). Contrarily, lowland species showed a puzzling distribution mismatching the expected areas, neither empirical data.

The use of flies synanthropy values derived from the Human Influence Index (HII) raster data set was a pivotal aspect of modeling; this variable improved the SDMs regardless of their synanthropic classification (Asynanthropic, Hemisynantropic, or Eusynanthropic). While the warm areas of low lands occupy a vast portion of the area surveyed (68.7%), the temperate and cold areas are restricted to the montane environments, being these a smaller fraction (31.3%) of the complete area surveyed. These differences in terms of size proportion may intrinsically influence the performance of the fly models obtained. In other words, the larger the area to evaluate, the lower the performance of the model and vice-versa. Furthermore, spatial distribution is not only related to climate variables but also other ecological interactions and bionomical attributes (besides synanthropy) not included in the input data to modeling. Analyzing potential distribution based on this methodology allowed identifying possible taxonomic inaccuracies and the lack of exhaustive collection, especially for lowlands species, while better performance was evidenced for species with montane and temperate distribution. The information here provided, contributes to biogeographical knowledge and will certainly serve as a contribution to the use of blow flies’ biological data for conservational and medical-legal purposes.

##  Supplemental Information

10.7717/peerj.10370/supp-1 Supplemental Information 1 Occurrence records of Calliphoridae species evaluated

10.7717/peerj.10370/supp-2 Supplemental Information 2 Potential distribution models in klm format

## Additional Information and Declarations

### Data Availability

The following information was supplied regarding data availability: Raw data are available as a Supplemental File.
